# Knowledge and awareness of tobacco user dental patients regarding effects of tobacco on oral health

**DOI:** 10.18332/tid/210415

**Published:** 2025-11-18

**Authors:** Umme Habiba, Rafaat Choudhury, Asadul Haque, Salma Sadia, Tahazid Tamannur, Mohibbul Haque, Nasrin Sultana, Shakila Jahan

**Affiliations:** 1 Department of Health Education, National Institute of Preventive and Social Medicine, Dhaka, Bangladesh; 2 Department of Microbiology and Mycology, National Institute of Preventive and Social Medicine, Dhaka, Bangladesh; 3 Department of Public Health and Informatics, Bangladesh Medical University, Dhaka, Bangladesh; 4 Department of Epidemiology, National Institute of Preventive and Social Medicine, Dhaka, Bangladesh; 5 Department of Dental Public Health, Dhaka Dental College, Dhaka, Bangladesh; 6 Department of Sociology, University of Dhaka, Dhaka, Bangladesh

**Keywords:** tobacco use, oral health, awareness, knowledge, dental patients

**Dear Editor,**

Tobacco use has become a serious concern in Bangladesh in both forms of consumption – smoked and smokeless^1,2^. Despite the fact that it is linked to periodontal disease, tooth loss, and oral cancer, awareness of its specific oral health consequences is limited. According to earlier research, even though the general health risks are established, knowledge of the oral effects is often inadequate^3,4^. A cross-sectional survey was conducted among 355 tobacco-user dental patients at Shaheed Suhrawardy Medical College and Dhaka Dental College Hospital from January to December 2022. During in-person interviews, information was gathered using a semi-structured questionnaire, employing convenience sampling. Knowledge scores were grouped into three categories based on total scores (good 7–10, average 4–6, poor 0–3). Data were analyzed using SPSS with descriptive statistics, chi-squared tests, and Pearson correlation.

Among the 355 participants, 63.1% were male and 52.1% were aged 38–57 years. Most (54.6%) used smoked tobacco, 41.1% used smokeless, and 4.3% used both. Peer influence (82%) was the main reason for initiation. Knowledge levels were: 67.9% good, 20.0% average, 12.1% poor (mean score=6.9 ± 2.38). Younger participants (18–37 years) had highest good knowledge (75.8%), followed by 67.9% in those aged 38–57 years, and 56.4% in those aged 58–77 years. Males had higher knowledge (76.3%) than females (53.4%). Mean awareness score was 5.0 ± 1.35. Females had slightly higher awareness than males (mean score: 5.19 vs 4.88, p=0.034). Knowledge correlated positively with awareness (r=0.133, p=0.012).

**Table 1:** Key demographic and behavioral factors associated with knowledge and awareness of tobacco’s effects on oral health

*Variable*	*Category*	*Percent*	*Key findings (%)*
**Age** (years)	18–37	35.2	Highest knowledge (75.8)
	38–57	52.1	Moderate knowledge (67.9)
	58–77	12.7	Lowest knowledge (56.4)
**Gender**	Male	63.1	Higher knowledge (76.3)
	Female	36.9	Lower knowledge (53.4)
**Educational level**	No formal education	25.6	Lowest knowledge (45.1)
	Primary	29.0	Moderate knowledge (62.1)
	Secondary	18.0	Highest knowledge (81.3)
**Tobacco consumption form**	Smoked tobacco	54.6	Higher knowledge (77.8)
	Smokeless tobacco	41.1	Lower knowledge (55.5)
**Peer influence**	Main reason for use	82.0	Significant factor for initiation
**Knowledge score**	Overall	-	Good knowledge (67.9)
**Awareness score**	Overall	-	Mean score: 5.0 ± 1.35
**Correlation (knowledge–awareness)**	-	-	Positive correlation (r=0.133, p=0.012)

In this study, we found that dental patients had good knowledge but limited awareness of oral health risks in Dhaka city. In line with other research^2^, we observed that peer influence was the main initiation factor. Women showed slightly higher awareness, while younger, educated participants had better knowledge. Since knowledge does not always change behavior, targeted interventions for older and less-educated groups are needed. However, the cross-sectional design restricts causal interpretation, and the hospital-based convenience sample may limit generalizability. Despite these limitations, the study highlights persistent gaps in oral health awareness among tobacco users. In conclusion, tobacco-using dental patients in Dhaka city have good knowledge of tobacco’s oral health effects, yet awareness remains inadequate. Tailored educational programs focusing on older adults, women, and less-educated groups are crucial to strengthen awareness and support cessation efforts.

## Data Availability

The data supporting this research are available from the authors on reasonable request.
